# Supplementary material for: Height-Age as An Alternative to Height-For-Age z-Scores to Assess the Effect of Interventions on Child Linear Growth in Low- and Middle-Income Countries
Source: Curr Dev Nutr. 2024 Oct 28;8(12):104495. doi: 10.1016/j.cdnut.2024.104495 (PMC11621485; doi:10.1016/j.cdnut.2024.104495)
Supplement: Multimedia component 1 [file mmc1.pdf]

Supplementary Material

Height-age as an alternative to height-for-age z-scores to assess the effect of interventions on child linear growth in low- and middle-income countries

Kelly M Watson, Alison SB Dasiewicz, Diego G Bassani, Chun-Yuan Chen, Huma Qamar, Karen M O'Callaghan, Daniel E Roth

Corresponding Author: Daniel E Roth (daniel.roth@sickkids.ca)

Table of Contents

1. Supplementary Methods 2
1.1 Calculating measures of precision for the PMB 2
2. Supplementary Results 4
2.1 Correlation model 4
2.2 Sensitivity analysis 5
References 6

1. Supplementary Methods

1.1 Calculating measures of precision for the PMB

Step 1: The standard deviation (SD) of the change in height-age during the intervention period for each group, denoted as $SDC_{0:1}$, was calculated using *Equation S1* (1).

Equation S1: $$SDC_{0:1} = \sqrt{SD_0^2 + SD_1^2 - (2 * Correlation * SD_0 * SD_1)}$$

Variable definitions, whereby SD_0 represents the group's mean height-age SD at baseline, SD_1 represents the group's mean height-age SD at end-line, and Correlation is the Pearson correlation (Pearson product-moment correlation coefficient) between a group's baseline (denoted '0') and end-line (denoted '1') height-age.

Step 1.1: We used individual participant data from the MDIG and BONUSKids studies (2, 3) to model the correlation between height-age at all possible combinations of starting and follow-up ages. This model was used to calculate a correlation value (input to Equation S1) for any study without individual-level data (provided the study population was similar to that of the MDIG trial cohort), based on children's age at baseline and end-line of the intervention period. Pearson correlation values from the length correlation matrix were used to develop the model of correlation coefficients (0, 3, 6, 9, 12, 15, 18, 21, 24 months)[1]. A simple linear regression model of the resulting Pearson correlations was used, with starting age and follow-up age as the two explanatory variables, as shown below. All analyses were performed in STATA software version 17 (STATA Corp LLC).

$$\text{Correlation} = \beta_0 + \beta_1(\text{starting age}) + \beta_2(\text{follow up age})$$

[1] It was of interest to include the birth measurement from the MDIG trial in the correlation matrix to maximize the number of observations included in the final model; however, height-age cannot be calculated at birth due to the truncation of age values in the WHO-LMS table at 0 days, which methods in this work did not address. Given that correlation coefficients are scale independent, it was expected that correlation values at all other time points would be similar between the length and height-age matrices as height-age is derived from length. This was confirmed by comparing height-age and length matrices. Thus, the length correlation matrix was used for modelling purposes.

Step 2: The $SDC_{0:1}$ and end-line sample size for each group were then used to calculate the pooled SD, standard error, and 95% confidence interval (CI) of the mean difference (intervention minus control) in the change in height-age (from baseline), using the method for two independent samples with a continuous outcome, assuming an alpha level of 5% (4).

Step 3: The upper and lower bounds calculated for the mean difference between group's change in height-age during the intervention period (as outlined in Step 2) were substituted into the numerator of the novel proportion of maximal benefit (PMB) metric to determine the 95% CI for the PMB.

2. Supplementary Results

2.1 Correlation model

The resulting model was:

Correlation = 0.797 + 0.016(starting age in months) - 0.004(follow up age in months)

Model output is presented in **Supplementary Table 1**. This model was used to determine the correlation value to input into *Equation S1* when calculating the standard deviation of the change in height-age ($SDC_{0:1}$) for studies where the correlation was unknown, as a necessary step to determine measures of precision for the PMB metric. For example, if the baseline of an intervention period was 6-months and the end-line was 12-months for a study where the correlation was unknown, the correlation value could be calculated from this model as: 0.797 + 0.016(6) - 0.004(12) = 0.845.

Supplementary Table 1. Linear regression output of Pearson correlation values between subsequent length measurements.

**Correlation model**	n	Regression coefficient	95% CI	p-value
Starting age, months	45	0.016	0.014, 0.018	< 0.001
Follow-up age, months	45	-0.004	-0.005, -0.003	< 0.001
Intercept	45	0.797	0.772, 0.821	< 0.001

2.2 Sensitivity analysis

Supplementary Table 2. Comparison of height-age estimates determined from mean LAZ/HAZ versus length/height.

Trial (*intervention duration*)	Group	Trial End-line			Follow-up[2,4]		
			Mean height-age (95% CI), d			Mean height-age (95% CI), d	
		n	Length-HA	LAZ-HA	*n*	Height-HA	HAZ-HA
MDIG (*0-6 months*)[1]	C	206	153 (148, 159)	153 (148, 158)	107	1248 (1207, 1291)	1249 (1207, 1291)
	I	210	156 (151, 161)	157 (152, 162)	114	1266 (1234, 1299)	1265 (1232, 1299)
DIVIDS (*0-6 months*)	C	638	NA[3]	118 (116, 121)	466	1347 (1309, 1385)	1356 (1335, 1377)
	I	620	NA[3]	122 (119, 124)	446	1351 (1313, 1390)	1370 (1349, 1392)

[1] Different primary end-line selected for this study (6 months) than the primary end-line reported in the MDIG trial (1 year).

[2] ‘Follow-up’ represents measurement of children after an elapsed period following cessation of the trial.

[3] Length data not available.

[4] The average age of children at the DIVIDS trial follow-up in both groups was 5 years, with a standard deviation of 1 year.

C, control group; CI, confidence interval; d, days; DIVIDS, Delhi Infant Vitamin D Supplementation; HA, height-age; HAZ, height-for-age z-score; I, intervention group; LAZ, length-for-age z-score; MDIG, Maternal Vitamin D for Infant Growth.

References

1. 16.1.3.2 Imputing standard deviations for changes from baseline [Internet]. The Cochrane Collaboration [cited 2021 Nov 29]. Available from: https://handbook-5-1.cochrane.org/chapter_16/16_1_3_2_imputing_standard_deviations_for_changes_from_baseline.htm.

2. Roth DE, Morris SK, Zlotkin S, Gernand AD, Ahmed T, Shanta SS, et al. Vitamin D Supplementation in Pregnancy and Lactation and Infant Growth. N Engl J Med. 2018;379(6):535-46.

3. O'Callaghan KM, Shanta SS, Fariha F, Harrington J, Mahmud AA, Emdin AL, et al. Effect of maternal prenatal and postpartum vitamin D supplementation on offspring bone mass and muscle strength in early childhood: follow-up of a randomized controlled trial. Am J Clin Nutr. 2022;115(3):770-80.

4. Confidence Interval for Two Independent Samples, Continuous Outcome [Internet]. Boston University School of Public Health2017 [cited 2022 May 2]. Available from: https://sphweb.bumc.bu.edu/otlt/mph-modules/bs/bs704_confidence_intervals/bs704_confidence_intervals5.html.
